# Dynamical system analysis of interacting dark energy in LRS Bianchi type I cosmology

**DOI:** 10.1038/s41598-023-40457-2

**Published:** 2023-08-26

**Authors:** Shivangi Rathore, S. Surendra Singh

**Affiliations:** Department of Mathematics, National Institute of Technology Manipur, Imphal, 795004 India

**Keywords:** Astronomy and planetary science, Mathematics and computing

## Abstract

This paper deals with an interacting dark energy (DE) model in Locally rotationally symmetric (LRS) Bianchi type I cosmological model with scalar field in the form of an exponential potential. We reduce the transformation equations to an independent system of ordinary differential equations by appropriate alteration of the variables to setup the complementary dynamical system and after that we also calculate the critical points of the system. We get six critical points when our coupling parameter is positive. And we get two critical points when our coupling parameter is negative. And dark energy behaves like a perfect fluid for all the critical points. And after that we analyzed all the critical points by calculating the eigenvalues of the Jacobian matrix and we find out that out of these eight critical points, six critical points are stable, which shows that our Universe is accelerating. And two (2) critical points are unstable. We also present the phase plot analysis.

## Introduction

Over the period of time, the variety of conceptual models that explain the fast evolution of our Universe has been developed by many researchers which help us to get the better understanding of our cosmos. For addressing the cosmic expansion of the Universe, the cosmological research community has confined two promising approaches: The first one is the characterization of dark energy, the most mysterious entity in the Universe. It has a negative pressure and positive energy density. According to the latest discoveries from the Wilkson Microwave Anisotropic Probe (WAMP)^1^ and other cosmological studies, the Universe contains $68.5 \%$ dark energy, $26.5\%$ dark matter and $5\%$ of baryonic matter. Both the cosmological constant and the equation of state parameter (EOS) $\omega =\frac{p}{\rho }$, where *p* is pressure and $\rho$ is energy density, can be used to describe dark energy. The Einstein-Hilbert action principle allows for an updated version of Einstein’s general relativity field equations, which are used in the second method of approach to describe the advancement of the Universe. A random function is used in this method in place of the matter Lagrangian. After that, these revised ideas become the most appealing challenges to observe the Universe’s fast expansion and the practical factors connected to dark energy.

Additionally, it has been suggested that the fundamental Einstein-Hilbert action is altered by the arbitrary function *f*(*R*). The *f*(*R*) gravity became a sufficient theory to explain the Universe’s early inflation and the late-time accelerated Universe also proposes a gravitational alternative for dark energy^2–9^. By combining the initial arbitrary function of the Ricci scalar *R* with the Lagrangian matter density $L_{m}$ in 2007, the *f*(*R*) gravity theory is reconstructed^10^. In 2011, Harko et al.^11^ come up with modified theory called the *f*(*R*, *T*) theory through the continuation of this coupling work where the gravitational component of the action depends on the Ricci scalar *R* as *f*(*R*) theories and also on trace *T*. The accelerated expansion of the Universe was done by developing an incredible amount of modified gravitational theories, including *f*(*G*) gravity, *f*(*R*, *G*) gravity, and *f*(*T*) gravity among others. Myrzakulov et al.^12^ have examined the inflation $f(R,\phi )$ theories of gravity where scalar field is associated with gravity. A brief overview of several *f*(*R*) gravity models for inflation in particular, Starobinsky-like inflation, has been provided by Sebastiani and Myrzakulov^13^. The most discussed explanation for analyzing the outcome of the Universe’s late-time accelerating expansion is explained by *f*(*R*, *T*) gravity theory. During the reconstruction of the *f*(*R*, *T*) gravity theory, there was also a phase change from a matter-dominated period to an accelerated phase^14^. Numerous cosmological models can be built using the *f*(*R*, *T*) gravity theory by selecting various matter sources.

To study the Universe’s accelerating expansion, the majority of researchers developed cosmological models with the perfect fluid matter. Recent observations indicate that the Universe is expanding faster than usual because of dark energy, an unknown kind of energy with a negative pressure. Light scalar fields, also known as quintessence, are the most basic representation of emerging dark energy. These quintessence-type dark energy models are highly recognized in the literature.

Scalar fields now play a significant part in the latest cosmological models of the premature cosmos^15,16^. An exponential scalar field dependency is a typical functional form for the self-interaction potential. Before researchers looked at the potential cosmological roles of exponential potentials, almost usually as a way to trigger a period of cosmological inflation. Scalar fields with exponential potentials might still have significant implications even if they are sufficiently abrupt to trigger an inflationary era, as per Copeland et al.^17^.

Here we take into account quintessence with an exponential potential

1 $$\begin{aligned} V(\phi )=V_{0}e^{-\lambda \phi } \end{aligned}$$

where $\lambda$ is dimensionless parameter and $V_{0}>0$.

Even though the Einstein equations seem straightforward and finding their exact solutions to any set of non-linear differential equations is highly challenging (and possibly impossible). Even if an analytical solution can be found, it will not be the only one of its kind; rather, it will be one of many^18^. In addition, there is concern about the stability of obtaining solutions. In this situation, dynamical systems are the way that allows us to associate asymptotic cosmological solutions with fundamental notation like saddle points and past and future attractors. The dynamical system is a set-up that is used to scrutiny being so uncomplicated to something as complicated as our Universe. In a dynamical system, we are recasting the cosmological equations and examining the dynamics of the Universe. This method enables the combination of analytical and numerical techniques to produce quantitative data on the models being investigated^19–21^. A dynamical system, also known as an ambient space system, is a mathematical framework that explains how the position of a point changes over time. Here, we address the system using a autonomous system of ordinary differential equations (ASODE). ASODE is a system of ordinary differential equations that does not explicitly confine on the time. Chingtham sonia and Surendra^22,23^ have used this technique to scrutiny models in the existence of a time-varying cosmological constant using different possibilities of *G* and $\rho _{\Lambda }$. And they also find out the critical points to inspect the stability and also the late time response of the premature cosmos.

Keeping in mind that the dynamics of the model with scalar fields are actively analyzed by using phase-space diagram. Stability and phase space assessment together is a comprehensive strategy to investigate potential inflationary behaviour.

Again, scrutiny has been made to determine the isotropy and homogeneity of the cosmos. It is thought that the geometry of the Universe was homogenous and isotropic at the end of the inflationary era, where Friedmann Lemaitre Robertson Walker (FLRW) models have a crucial role in depicting both spatially isotropic and homogeneous cosmos. However, the theoretical case and the aberration discovered in the CMB give attestation that an anisotropic phase later termed an isotropic one exists. Soon after the release of the Plank Probe data, it is assumed that the premature Universe wasn’t perfectly uniform. As a result, the idea that the Universe is inhomogeneous and anisotropic has become more and more popular when developing cosmological models with an anisotropic background. Thus, models of the Bianchi type are vital for explaining the premature cosmos with the anisotropic context. Locally rotationally symmetric (LRS) refers to the group of cosmological models that consider rotational symmetry at each point in space. These models examine the behavior of Universe on a large scale. And these models are simplified version of general cosmological models. In LRS Bianchi type I models, we consider that our Universe is homogenous and anisotropic on large scale but allows for local rotations. And it is one of the moat useful tools for studying the evolution and dynamics of the Universe. Many academics have looked at the homogeneous and anisotropic Bianchi type models for exploring the cosmic advancement of the premature Universe due to the analytical challenges in researching the inhomogeneous models. Bianchi space-times occur in 9 distinct forms in literature (I–IX). Since it is the more articulately spatially homogenous and anisotropic, we will focus on Bianchi type-I space-time in this case. It is also referred to as the FLRW flat metric’s prompt generalization with various scale factors in each spatial direction. In the context of a viscous fluid, the essence of the Bianchi type-I model has been examined. Viscosity has been found to affect the qualitative conduct of solutions closer to singularity without impacting the early big bang singularity itself^24^.

The elementary approach to acknowledging the dynamics of a system near a critical point is to apply the idea of linear stability theory. When we scrutiny the stability of a critical point by evaluating the eigenvalues of the Jacobian matrix at the critical point.

Models entrench dark energy (DE) interacting with dark matter (DM) or any other exotic matter components have appreciated over the last few years. These interactive DE models can satisfactorily explain a number of cosmological mysteries, including the cosmic age problem, cosmic coincidence, and phantom crossing^25–29^.

This paper deals with the dynamics of interaction between DE and DM of the Bianchi type I model. To acknowledge the mechanics of DE, it may be imperative to address the interplay between DE and DM. However, it won’t be feasible to determine the exact form of the interaction from first principles because the nature of these two components is still hidden. A particular coupling must be presupposed or deduced from phenomenological conditions. Furthermore, it seems sense to think about how the dark components will inevitably interact within the context of field theory. A mechanism to address the coincidence issue can be provided by an adequate interplay between DE and DM. DM and DE, the two main elements of the cosmic composition^30–33^ at the moment, typically interact in a phenomenological manner. The convertibility of energy density in the dark sector is provided by the interaction term. The purpose of selecting such a complicated system is to see if a strange model might adequately account for the overall evolution of the Universe. We examine the dynamics in the phase space related to this situation around both hyperbolic and non-hyperbolic critical points because the field equations are complex. In a dynamical system, critical points are the points where the system may change its behavior. A critical point is hyperbolic if the linearization of system of equations has eigenvalues with non-zero real parts otherwise it is referred to as the non-hyperbolic critical point. For more understanding, let’s consider a two-dimensional dynamical system ${\dot{x}}=f(x,y)$ and ${\dot{y}}=g(x,y)$ The critical point $(x_{0},y_{0})$ is hyperbolic if the Jacobin matrix of the system at this critical point has eigenvalues with non-zero real parts. Otherwise these critical points are non-hyperbolic critical points. Hyperbolic critical points play an important role in studying the dynamics of the system as they determine the behavior and stability properties of the system. We investigate the characteristics of critical points by taking into account the eigenvalues of a first-order perturbed matrix close to the critical points. Erstwhile, similar kind of dynamic system analysis is done by Dirac-Born-in (DBI) field gravity theory^34^ and in Brane Scenarion^35^. Critical points are calculated and their stability is rationalized in the DBI model. In addition to the inquiry described above, the classical stability of the Brane gravity model, which is based mostly on higher-dimensional theories have been investigated. In the present LRS Bianchi type I cosmological model, the transformation equations are converted to the autonomous system of equations by the appropriate alternation of the variables. And we also analyse the linear and classical adherence of the model.

In this work, we present the study in different sections. In section “The basic field equations”, we discuss some basic equations and section “Examination of phase-space and critical points” is all about the phase-space and critical points examination of our autonomous system. We discuss the stability of the model in sections “Stability analysis of our cosmological model” and “Critical points and cosmological significance”, we discuss the cosmological significance of the model based on the critical points. And at the end, we summarized and conclude our paper in section “Conclusion”.

## The basic field equations

The Hilbert-Einstein action is given by

2 $$\begin{aligned} S = \int \sqrt{-g}\bigg (-\frac{1}{2}(\nabla \phi )^{2} -V(\phi )\bigg )d^{4}x \end{aligned}$$

where $$(\nabla \phi )^{2} = g^{\mu \nu }\partial _{\mu }\partial _{\nu }\phi $$.

The Lagrangian is

3 $$\begin{aligned} L_{\phi } = -\frac{1}{2}(\nabla \phi )^{2} - V(\phi ) \end{aligned}$$

We assume the homogenous LRS Bianchi type I metric as

4 $$\begin{aligned} ds^{2} = dt^{2} - A^{2}_{1}dx^{2} - B^{2}_{1}\left( dy^{2}+dz^{2}\right) \end{aligned}$$

where $A_{1}$ and $B_{1}$ are the function of cosmic time *t*. The extensions of three-dimensional spaces in the *x*, *y* and *z* directions are represented by scale factors $A_{1}(t)$ and $B_{1}(t)$ respectively. The corresponding Hubble parameters in each direction are described as

5 $$\begin{aligned} H_{i} = \frac{\dot{a_{i}}}{a_{i}}, i=1,2. \end{aligned}$$

with relation to cosmic time *t*, an overhead dot indicates a differential. Hubble parameter *H*, which is generally characterized as

6 $$\begin{aligned} H = \frac{{\dot{a}}}{a} = \frac{1}{3}\bigg (\frac{\dot{A_{1}}}{A_{1}}+\frac{2\dot{B_{1}}}{B_{1}}\bigg ) = \frac{1}{3}(H_{1}+2H_{2}) \end{aligned}$$

Here, we’ll suppose that $A_{1} \propto B^{n}_{1}$ where $n(n \ne 0,1)$ is an increasing constant. One can determine the relationship between the directional Hubble’s parameter and the generalized hubble’s parameter by using equation 6.

7 $$\begin{aligned} H_{1} = nH_{2}=\bigg (\frac{3n}{n+2}\bigg )H \end{aligned}$$

The Einstein’s field equations for Bianchi type I metric exist as

8 $$\begin{aligned}{} & {} 2\frac{\dot{A_{1}}\dot{B_{1}}}{A_{1}B_{1}} + \bigg (\frac{\dot{B_{1}}}{B_{1}}\bigg )^{2} = (\rho _{\gamma } + \rho _{\phi }) \end{aligned}$$

9 $$\begin{aligned}{} & {} 2\frac{\ddot{B_{1}}}{B_{1}}+\bigg (\frac{\dot{B_{1}}}{B_{1}}\bigg )^{2}= - (p_{\gamma }+p_{\phi }) \end{aligned}$$

10 $$\begin{aligned}{} & {} \frac{\ddot{A_{1}}}{A_{1}}+\frac{\ddot{B_{1}}}{B_{1}}+\frac{\dot{A_{1}}\dot{B_{1}}}{A_{1}B_{1}}=-\left( p_{\gamma }+p_{\phi }\right) \end{aligned}$$

For this particular scalar field, the energy density and pressure are

11 $$\begin{aligned} \rho _{\phi }= & {} \frac{1}{2}{\dot{\phi }}^{2}+V(\phi ) \end{aligned}$$

12 $$\begin{aligned} p_{\phi }= & {} \frac{1}{2}{\dot{\phi }}^{2}-V(\phi ) \end{aligned}$$

After using equation (7)–(12), the outcomes of the Friedmann and Raychaudhuri equations are given as

13 $$\begin{aligned} H^{2}= & {} \frac{(n+2)^{2}}{9(2n+1)}\bigg (\rho _{\gamma }+\frac{1}{2}{\dot{\phi }}^{2}+V(\phi )\bigg ) \end{aligned}$$

14 $$\begin{aligned} {\dot{H}}= & {} -\frac{(n+2)^{2}}{6(2n+1)}(\rho _{\gamma }+p_{\gamma }+{\dot{\phi }}^{2}) \end{aligned}$$

Here, we assume the Bianchi type I cosmos which contain a fluid with baryotropic equation of state $p_{\gamma }=(\gamma -1)\rho _{\gamma }$ where $\gamma$ is a constant and the value of $\gamma \in [0,2]$, for example for radiation the value of $\gamma$ is $\frac{4}{3}$ and for dust the value of $\gamma$ is 1.

For individual dark component, the energy balance equations are

15 $$\begin{aligned} {\dot{\rho }}_{\gamma }+3H\rho _{\gamma }=Q \end{aligned}$$

and

16 $$\begin{aligned} {\dot{\rho }}_{\phi }+3H(1+\omega _{\phi })\rho _{\phi }= -Q \end{aligned}$$

where *Q* is the interaction term that is related to energy transfer between Dark energy (DE) and Dark matter (DM). The value of *Q* is either positive or negative, it depends on the transfer of energy. When the energy transfers from dark energy to dark matter, then the value of *Q* is positive. And when the energy transfers from dark matter to dark energy the value of *Q* is negative. Although, here, we assume that the value of *Q* does not change its sign over the Universe transformation. For the shake of convincing, we assume the value of $Q=\alpha H \rho _{\gamma }$ where $\alpha$ is the coupling constant and we assume the value $\alpha$ is very small.

After using Eqs. (11) and (12) in the continuity equation (16), the modified Klein-Gordon equation is

17 $$\begin{aligned} \ddot{\phi }+3H {\dot{\phi }}+\frac{dV(\phi )}{d\phi }= -\frac{Q}{{\dot{\phi }}} \end{aligned}$$

Due to the complex nature of the evolution equation, the analytical solutions are not feasible. We will therefore integrate the evolution equations into a self-contained dynamical system to gain a qualitative interpretation of the cosmic behavior. The change in variables is the traditional way to talk about the stability of the systems. Such a transformation is advantageous in the exponential potential scenario because it converts the class of solutions to critical points of the new equations. To perform this task, we instigate new dimensionless variables

18 $$\begin{aligned} x^{2}= & {} \frac{(n+2)^{2}}{9(2n+1)}\bigg (\frac{{\dot{\phi }}^{2}}{2H^{2}}\bigg ) \end{aligned}$$

19 $$\begin{aligned} y^{2}= & {} \frac{(n+2)^{2}}{9(2n+1)}\bigg (\frac{V(\phi )}{H^{2}}\bigg ) \end{aligned}$$

20 $$\begin{aligned} \Omega _{\gamma }= & {} \frac{(n+2)^{2}}{9(2n+1)}\bigg (\frac{\rho _{\gamma }}{H^{2}}\bigg ) \end{aligned}$$

For Bianchi type-I Universe, the number of e-folding is given as $N=\frac{1}{3}ln \left( AB^{2}\right) =\left( \frac{n+2}{3}\right) lnB=\left( \frac{n+2}{3n}\right) lnA$ with the expectation that such a system will exhibit accelerated expansion. The evolution equations can be expressed as an autonomous system for potential $V(\phi )$ as follows

21 $$\begin{aligned} \frac{dx}{dN}= & {} -3x+ \frac{3\sqrt{2n+1}}{\sqrt{2}(n+2)}\lambda y^{2}+\left( 1-x^{2}-y^{2}\right) \bigg (\frac{3\gamma x}{2}-\frac{\alpha }{2x} \bigg )+3x^{3} \end{aligned}$$

22 $$\begin{aligned} \frac{dy}{dN}= & {} -\lambda \frac{3\sqrt{2n+1}}{\sqrt{2}(n+2)}xy + \frac{3}{2}y \left[ {\gamma \left( 1-x^{2}-y^{2}\right) +2x^{2}}\right] \end{aligned}$$

23 $$\begin{aligned} \frac{d\Omega _{\gamma }}{dN}= & {} \Omega _{\gamma }\left[ (\alpha -3)+3\gamma \left( 1-x^{2}-y^{2}\right) +6x^{2}\right] \end{aligned}$$

To analyze the system of equations in Eqs. (21)–(23) we have to first equate them equal to zero to get the critical points. After that, we examine the stability of first-order equations by perturbing them near the critical points. The interaction used to construct the preceding autonomous system of ODE is assumed to be in the form $Q=\alpha H \rho _{\gamma }$. Additionally, we obtain the density parameter for the DM using the standardized variables in the first modified Friedmann equation as

24 $$\begin{aligned} \Omega _{\gamma }=1-x^{2}-y^{2} \end{aligned}$$

Because of the energy condition, $0< \Omega _{\gamma } < 1$, hence for any fixed value of $\Omega _{\gamma }$, the value of (*x*, *y*) lies on the circle $x^{2}+y^{2}=1-\Omega _{\gamma }$. However, for the autonomous system’s (21)–(23), phase space $(x,y,\Omega _{\gamma })$ has the form of a paraboloid $(x^{2}+y^{2}+\Omega _{\gamma }=1)$ bounded by $\Omega _{\gamma } = 0$ and $\Omega _{\gamma } = 1$. Hence phase space is finite.

Newly defined variables can be used to express the equation of state parameter $\omega _{\phi }$ and the density parameter $\Omega _{\phi }$, which are cosmological parameters associated with scalar fields.

25 $$\begin{aligned} \omega _{\phi }= & {} \frac{2x^{2}}{x^{2}+y^{2}} \end{aligned}$$

26 $$\begin{aligned} \Omega _{\phi }= & {} \frac{3(2n+1)}{(n+2)^{2}}(x^{2}+y^{2}) \end{aligned}$$

and

27 $$\begin{aligned} \omega _{Tot}= \frac{p}{\rho }=\frac{p_{\phi }+p_{\gamma }}{\rho _{\phi }+\rho _{\gamma }} = x^{2}-y^{2}+(\gamma - 1)\Omega _{\gamma } \end{aligned}$$

Subsequently, we have $x^{2}+y^{2}+\Omega _{\gamma } = 1$. Hence, the explicit form of the deceleration parameter is

28 $$\begin{aligned} q = \frac{d}{dt}\bigg (\frac{1}{H}\bigg )-1 = -1 - \frac{{\dot{H}}}{H^{2}}= -1+\frac{3}{2}\gamma \left( 1-x^{2}-y^{2}\right) +3x^{2} \end{aligned}$$

## Examination of phase-space and critical points

A dynamical system is a potent tool to study cosmic evolution, where the exact solution could not be found due to the complicated system. Non-linear systems of differential equations are the dynamical systems that are most frequently found in cosmological systems. Here the dynamical system is also non-linear. Now we initially examine the critical points of the dynamical system (21)–(23) to access the dynamics of the Universe. Accordingly, we will plot the phase and evolutionary diagrams. Because of that first we have to calculate the critical points of the dynamical system and after that we linearize our model around the critical points. For the positive coupling parameter $\alpha$, we get six critical points:

- Critical points *A* ,*B* = $$(\pm 1,0,0)$$
- Critical points *C*,*D* = $$\bigg (\frac{\lambda \sqrt{2n+1}}{\sqrt{2}(n+2)},\pm \sqrt{1-\frac{\lambda ^{2}(2n+1)}{2(n+2)^{2}}},0\bigg )$$
- Critical point *E*,*F* = $\bigg (-\frac{(\alpha -3)(n+2)}{3\sqrt{2}\lambda \sqrt{2n+1}},\pm \sqrt{1-\frac{(n+2)^{2}(\alpha -3)^{2}}{18 \lambda ^{2(2n+1)}}},\frac{(3-\alpha )}{\gamma }\bigg [\frac{1}{3}-\frac{(3-\alpha )(n+2)^{2}}{9\lambda ^{2}(2n+1)}\bigg ]\bigg )$ Even though, there are two critical points for the negative coupling parameter.
- Critical point *G* , *H* = $\bigg (\pm \sqrt{\frac{-\alpha }{3}},0,\frac{\alpha +3}{3\gamma }\bigg )$ where $\alpha \in (-3,0)$. Tables 1 and 2 display these critical points along with the essential physical parameters at those points.

**Table 1:** The critical points and the related parameters at these points when $\alpha$ is positive.

Point	*x*	*y*	${\Omega }_{\gamma }$	${\omega }_{\phi }$	${\Omega }_{\phi }$	*q*	${\omega }_{(Tot)}$
*A*	1	0	0	2	$\frac{3(2n+1)}{(n+2)^{2}}$	2	1
*B*	-1	0	0	2	$\frac{3(2n+1)}{(n+2)^{2}}$	2	1
*C*	$\frac{\lambda \sqrt{2n+1}}{\sqrt{2}(n+2)}$	$\sqrt{1-\frac{\lambda ^{2}(2n+1)}{2(n+2)^{2}}}$	0	$\frac{\lambda ^{2}(2n+1)}{(n+2)^{2}}$	$\frac{3(2n+1)}{(n+2)^{2}}$	$-1+\frac{3\lambda ^{2}(2n+1)}{2(n+2)^{2}}$	$\frac{\lambda ^{2}(2n+1)}{(n+2)^{2}}-1$
*D*	$\frac{\lambda \sqrt{2n+1}}{\sqrt{2}(n+2)}$	$-\sqrt{1-\frac{\lambda ^{2}(2n+1)}{2(n+2)^{2}}}$	0	$\frac{\lambda ^{2}(2n+1)}{(n+2)^{2}}$	$\frac{3(2n+1)}{(n+2)^{2}}$	$-1+\frac{3\lambda ^{2}(2n+1)}{2(n+2)^{2}}$	$\frac{\lambda ^{2}(2n+1)}{(n+2)^{2}}-1$
*E*	$\frac{-(\alpha -3)(n+2)}{3\sqrt{2}\lambda \sqrt{2n+1}}$	$\sqrt{1-\frac{(n+2)^{2}(\alpha -3)^{2}}{18\lambda ^{2}(2n+1)}}$	$\frac{(3-\alpha )}{\gamma }\bigg [\frac{1}{3}-\frac{(3-\alpha )(n+2)^{2}}{9\lambda ^{2}(2n+1)}\bigg ]$	$\frac{(\alpha -3)^{2}(n+2)^{2}}{9\lambda ^{2}(2n+1)}$	$\frac{3(2n+1)}{(n+2)^{2}}$	$-1+\frac{(\alpha -3)^{2}(n+2)^{2}}{6\lambda ^{2}(2n+1)}$	$-1+\frac{(\alpha -3)^{2}(n+2)^{2}}{9\lambda ^{2}(2n+1)}+\frac{(\gamma -1)(3-\alpha )}{\gamma }\bigg [\frac{1}{3}-\frac{(3-\alpha )(n+2)^{2}}{9\lambda ^{2}(2n+1)}\bigg ]$
*F*	$-\frac{(\alpha -3)(n+2)}{3\sqrt{2}\lambda \sqrt{2n+1}}$	$-\sqrt{1-\frac{(\alpha -3)^{2}(n+2)^{2}}{18\lambda ^{2}(2n+1)}}$	$\frac{(3-\alpha )}{\gamma }\bigg [\frac{1}{3}-\frac{(3-\alpha )(n+2)^{2}}{9\lambda ^{2}(2n+1)}\bigg ]$	$\frac{(\alpha -3)^{2}(n+2)^{2}}{9\lambda ^{2}(2n+1)}$	$\frac{3(2n+1)}{(n+2)^{2}}$	$-1+\frac{(\alpha -3)^{2}(n+2)^{2}}{6\lambda ^{2}(2n+1)}$	$-1+\frac{(\alpha -3)^{2}(n+2)^{2}}{9\lambda ^{2}(2n+1)}+\frac{(\gamma -1)(3-\alpha )}{\gamma }\bigg [\frac{1}{3}-\frac{(3-\alpha )(n+2)^{2}}{9\lambda ^{2}(2n+1)}\bigg ]$

**Table 2:** The critical points and relevant parameters at these points when $-3< \alpha < 0$.

Points	*x*	*y*	$\Omega _{\gamma }$	$\omega _{\phi }$	$\Omega _{\phi }$	*q*	$\omega _{(Tot)}$
*G*	$\sqrt{\frac{-\alpha }{3}}$	0	$\frac{\alpha +3}{3 \gamma }$	2	$-\frac{\alpha (2n+1)}{(n+2)^{2}}$	$-1-\alpha +\frac{3\gamma }{2} \left( 1+\frac{\alpha }{3}\right)$	$-1-\frac{2\alpha }{3}+\gamma \left( 1+\frac{\alpha }{3}\right)$
*H*	$-\sqrt{\frac{-\alpha }{3}}$	0	$\frac{\alpha +3}{3 \gamma }$	2	$-\frac{\alpha (2n+1)}{(n+2)^{2}}$	$-1-\alpha +\frac{3\gamma }{2} \left( 1+\frac{\alpha }{3}\right)$	$-1-\frac{2\alpha }{3}+\gamma \left( 1+\frac{\alpha }{3}\right)$

From Table 1, we observe that the critical points *A* and *B* exist always while the critical points *C* and *D* exist only for $\lambda ^{2} < \frac{2(n+2)^{2}}{(2n+1)}$. Also, the critical points *A*, *B*, *C* and *D* exhibit only the DE component (DM is absent). While critical points *E* and *F* exist for $18\lambda ^{2}(2n+1)-(n+2)^{2}(\alpha -3)^{2}> 0$ and proportionate a blend of DM and DE with the ratio of two energy densities $r= \frac{\Omega _{\gamma }}{\Omega _{\phi }}= \frac{(3-\alpha )(n+2)^{2}}{3\gamma (2n+1)}\bigg [\frac{1}{3}-\frac{(3-\alpha )(n+2)^{2}}{9\lambda ^{2}(2n+1)}\bigg ]$. For all the critical points, DE behaves like a perfect fluid. Moreover, in Table 2, we observe that *G* and *H* are the unions of DM and DE with the ratio of their density parameter $\frac{\Omega _{\gamma }}{\Omega _{\phi }}=\bigg (-1-\frac{3}{\alpha }\bigg )\bigg (\frac{(n+2)^{2}}{3(2n+1)}\bigg )$.

## Stability analysis of our cosmological model

We will now explain the stability of the autonomous system’s (21)–(23) critical points, which are listed in Tables 1 and 2. Focus on first perturbation close to the critical points. The eigenvalues of the matrix which is shown in Tables 3 and 4 below, must be investigated to understand the characteristics of the critical points.

**Table 3:** Eigenvalues of the linearized matrix for the autonomous system’s critical points when $\alpha$ is positive.

Points	$\lambda _{1}$	$\lambda _{2}$	$\lambda _{3}$
*A*	$3+\alpha$	$3-\frac{3\lambda \sqrt{2n+1}}{\sqrt{2}(n+2)}$	$3+\alpha$
*B*	$3+\alpha$	$3+\frac{3\lambda \sqrt{2n+1}}{\sqrt{2}(n+2)}$	$3+\alpha$
*C*	$(\alpha -3)+\frac{3\lambda ^{2}(2n+1)}{(n+2)^{2}}$	$\frac{9\lambda ^{2}(2n+1)}{(n+2)^{2}}\bigg [\frac{\lambda ^{2}(2n+1)}{2(n+2)^{2}}-1\bigg ]$	$\frac{9\lambda ^{2}(2n+1)}{2(n+2)^{2}}-4$
*D*	$(\alpha -3)+\frac{3\lambda ^{2}(2n+1)}{(n+2)^{2}}$	$\frac{9\lambda ^{2}(2n+1)}{(n+2)^{2}}\bigg [\frac{\lambda ^{2}(2n+1)}{2(n+2)^{2}}-1\bigg ]$	$\frac{9\lambda ^{2}(2n+1)}{2(n+2)^{2}}-4$
*E*	$(\alpha -3)+\frac{(\alpha -3)^{2}(n+2)^{2}}{3\lambda ^{2}(2n+1)}$	3	$-2$
*F*	$(\alpha -3)+\frac{(\alpha -3)^{2}(n+2)^{2}}{3\lambda ^{2}(2n+1)}$	$-\frac{4}{9}$	$-1$

**Table 4:** Eigenvalues of the linearized matrix for the critical points when $\alpha \in (-\,3,0)$.

Points	$\lambda _{1}$	$\lambda _{2}$	$\lambda _{3}$
*G*	$-\frac{9}{2}-\frac{7\alpha }{2}+\frac{\gamma (3+\alpha )}{2}$	$\frac{3\sqrt{3}\lambda \sqrt{2n+1}}{\sqrt{2}\sqrt{\alpha }(n+2)}+\frac{1}{2} \left[ \gamma (3+\alpha )-2\alpha \right]$	$(\gamma -1)(\alpha +3)$
*H*	$-\frac{9}{2}-\frac{7\alpha }{2}+\frac{\gamma (3+\alpha )}{2}$	$\frac{3\sqrt{3}\lambda \sqrt{2n+1}}{\sqrt{2}\sqrt{\alpha }(n+2)}+\frac{1}{2} \left[ \gamma (3+\alpha )-2\alpha \right]$	$(\gamma -1)(\alpha +3)$

**Figure 1:**
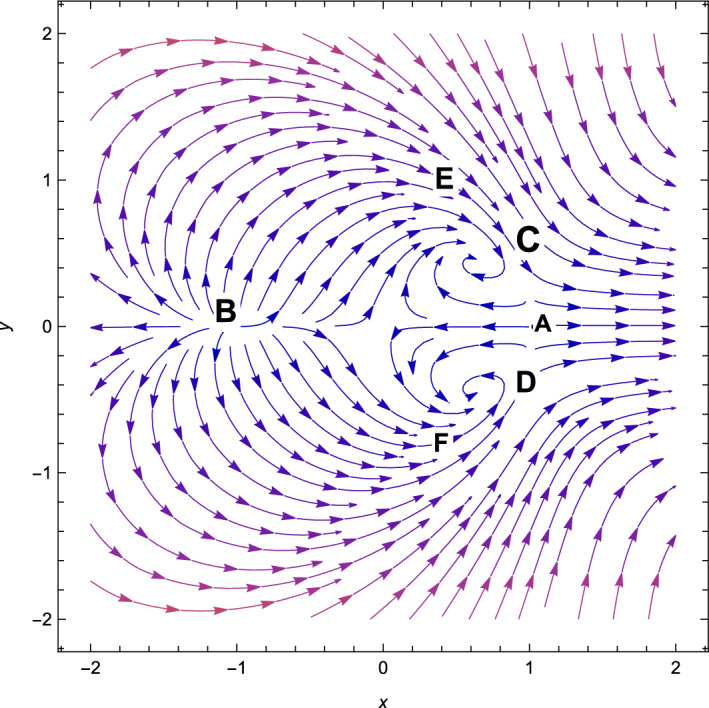
The phase plot when $(n=2,\alpha = 0.001,\lambda = 2.7, \gamma =4/3)$.

**Figure 2:**
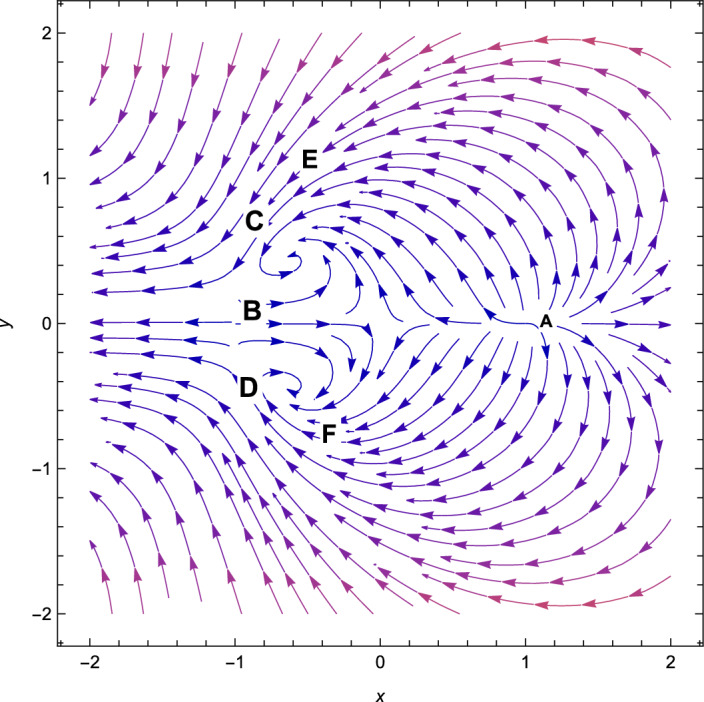
Phase plot when $(n=2,\alpha =0.001 , \lambda = -\,2.70, \gamma =4/3)$.

**Figure 3:**
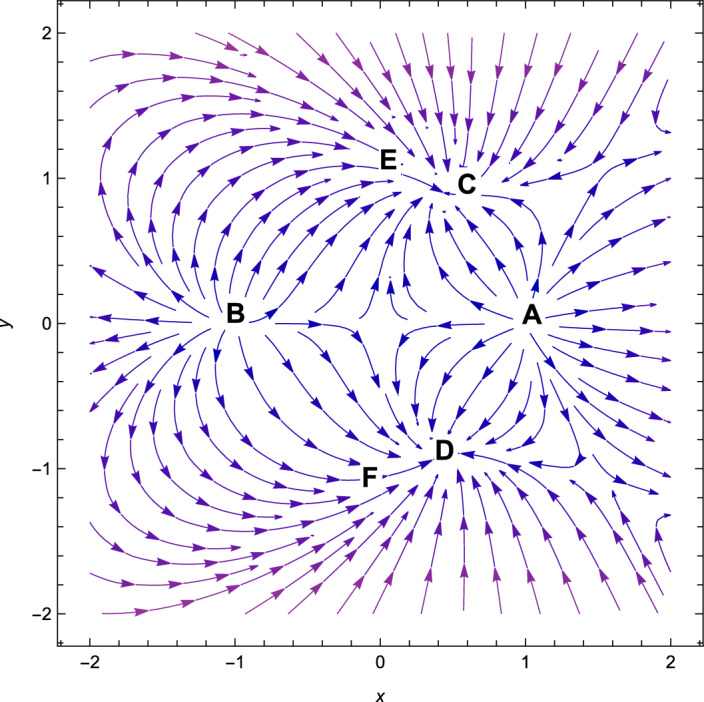
Phase plot when $n=2,\alpha =0.001,\lambda =1.10,\gamma =4/3$ in the plane $(x,y,\Omega _{\gamma })$.

**Figure 4:**
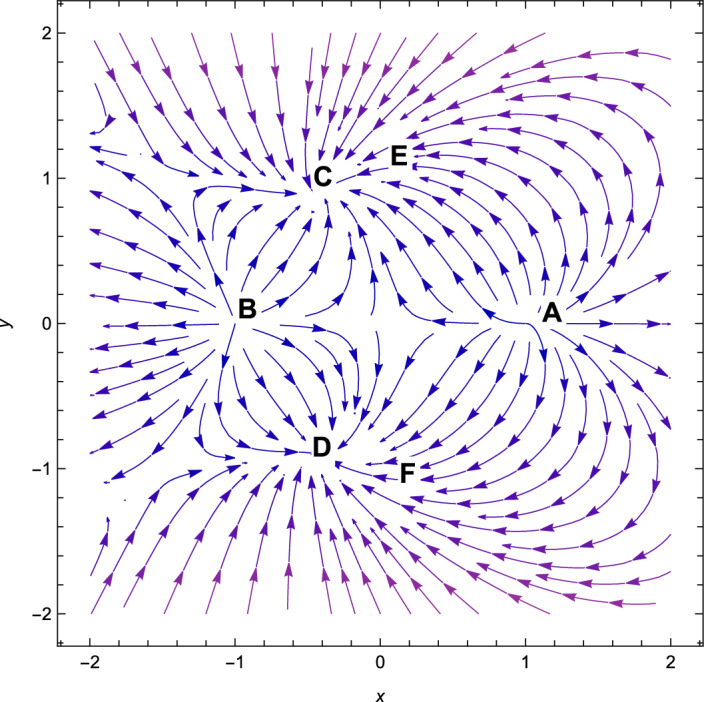
Phase plot when $n=2,\alpha =0.001,\lambda =-\,1.10,\gamma =4/3$.

**Figure 5:**
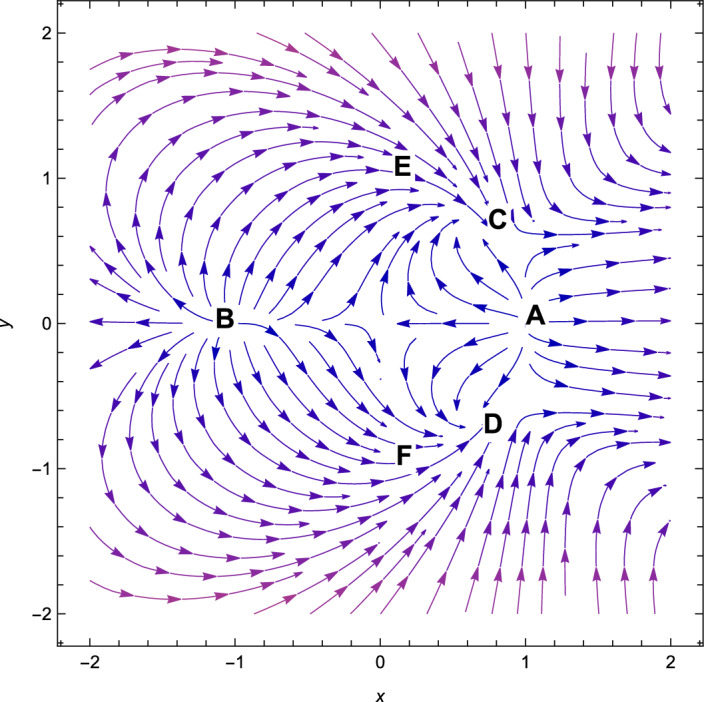
Phase plot when $n=2,\alpha =0.001,\lambda =1.90,\gamma =4/3$.

**Figure 6:**
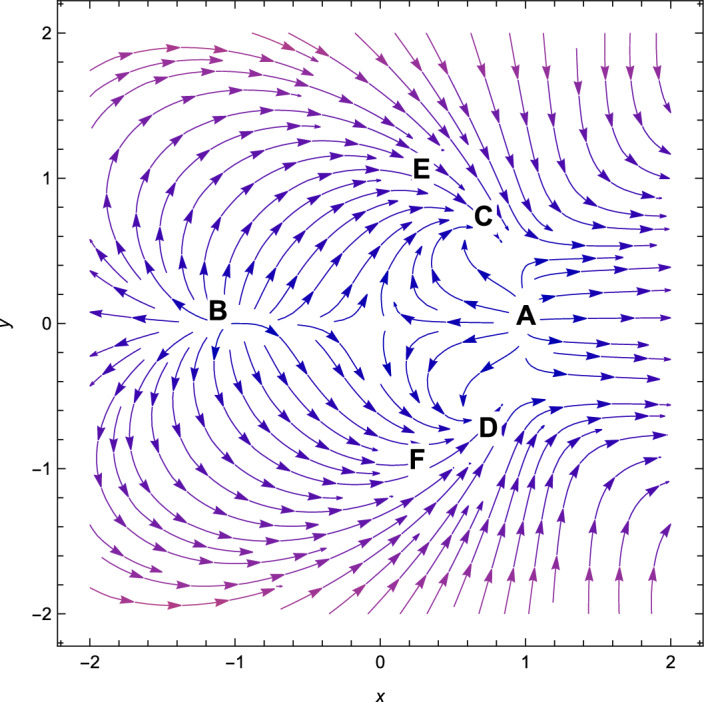
Phase plot for $n=2,\alpha =0.001,\lambda =2.10,\gamma =4/3$.

**Figure 7:**
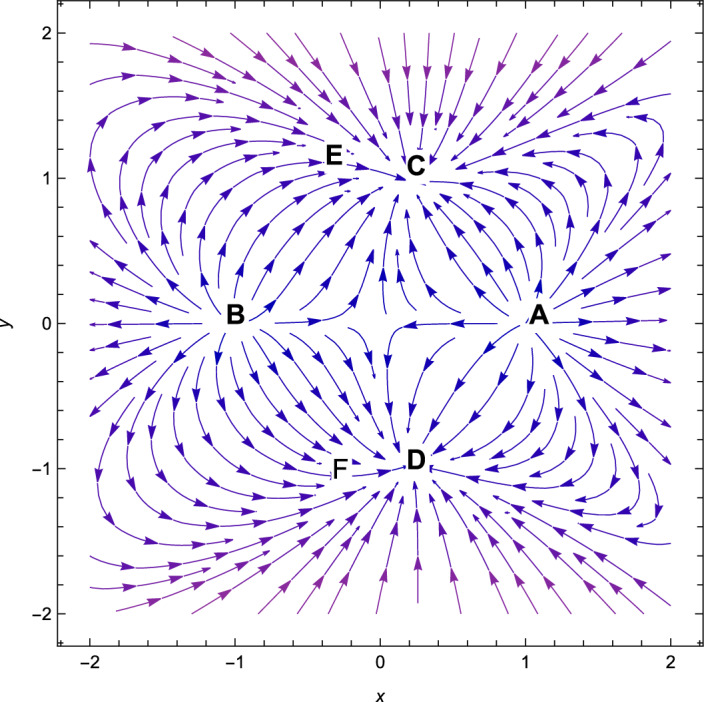
Phase plot when $(n=2,\alpha =0.001,\lambda =0.5,\gamma =4/3)$.

**Figure 8:**
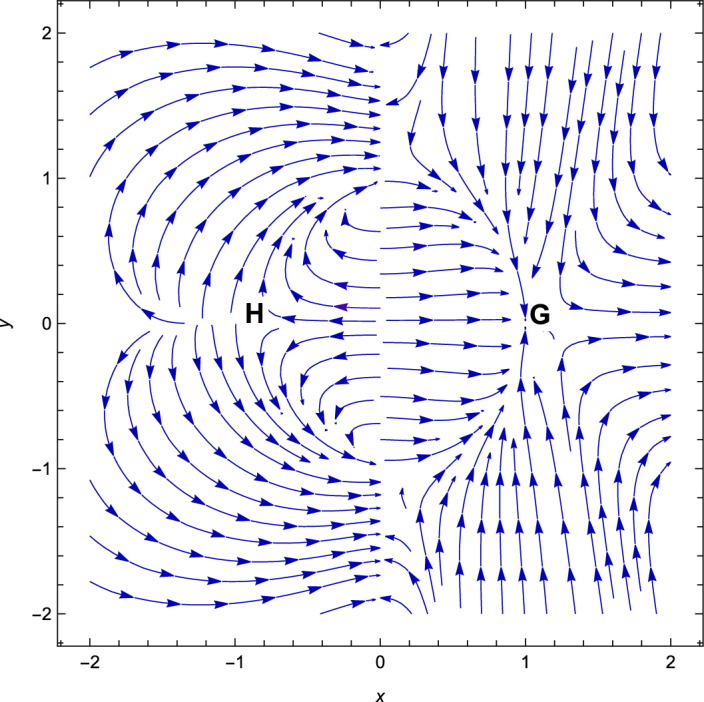
Phase plot when $(n=2, \alpha = -\,2.9, \lambda =2.7, \gamma =4/3)$.

Based on observation, we can see that from Table 3, *A* is non-hyperbolic when the value of $\lambda =\frac{\sqrt{2}(n+2)}{\sqrt{2n+1}}$, otherwise, it is hyperbolic. However, when the value of $\lambda =-\,\frac{\sqrt{2}(n+2)}{\sqrt{2n+1}}$, the critical point *B* is non-hyperbolic otherwise it is hyperbolic. The critical point *A* is the saddle-node when the value of $\lambda > \frac{\sqrt{2}(n+2)}{\sqrt{2n+1}}$(see Fig. 1) and the critical point *B* is the saddle-node when the value of $\lambda < -\frac{\sqrt{2}(n+2)}{\sqrt{2n+1}}$(see Fig. 2) otherwise for $\lambda < \frac{\sqrt{2}(n+2)}{\sqrt{2n+1}}$, *A* is unstable node (Fig. 2) and *B* is unstable node when the value of $\lambda > \frac{\sqrt{2}(n+2)}{\sqrt{2n+1}}$(see Fig. 1). For the critical points *A* and *B*, there are no accelerating phases of the cosmos. Additionally, these two critical points indicate notions in which matter does not exist in the Universe. They are just solutions that are dominated by kinetic energy.

However, *C* and *D* are identical in every way and are hyperbolic character. If $0< \alpha < 3-\frac{3\lambda ^{2}(2n+1)}{(n+2)^{2}}$, these two critical points are stable solutions. And these critical points also behave like attractor solutions when $\lambda ^{2} < \frac{(n+2)^{2}}{(2n+1)}$. While these critical points behave like saddle nodes for $\frac{3(n+2)^{2}}{(2n+1)}< \lambda ^{2} < \frac{6(n+2)^{2}}{(2n+1)}$. Near the critical points *C* and *D*, there exists an accelerating Universe when $\lambda ^{2} < 2$.

Critical points *E* and *F* are identical in every way as well. These critical points are the combination of both DM and DE. Depending on the value of $\alpha$, they correspond to their solution in accelerating and decelerating phase of the Universe.

For the negative coupling parameter, we obtain two critical points *G* and *H* which are shown in Table 4. These critical points are non-hyperbolic. Critical points *G* and *H* are always non-hyperbolic because they are the critical points when the value of the decoupling parameter is negative viz. $-3$. And when we calculate the eigenvalues corresponding to these critical points, we get a degenerate eigenvalues. When we get degenerate eigenvalue corresponding to a critical point, then we refer to that critical point as a non-hyperbolic critical point. At these critical points, the solution is dominated by both the component which is DE and DM. Near these critical points, there is no accelerating phase of the Universe.

Consequently, the followings can be said to sum up our main findings.

- The kinetic energy leading solutions (points *A* and *B* in Table 1) are invariably unstable points for all $\lambda$ and $\alpha > 0$. In particular, for $\lambda \in (-\frac{\sqrt{2}(n+2)}{\sqrt{2n+1}},\frac{\sqrt{2}(n+2)}{\sqrt{2n+1}})$ and always decelerating $(q = 2)$, *A* and *B* signify source or repeller (unstable node) (see Figs. 3, 4 and 7). Figure 7 shows the projection of phase plot to a given plane $(x,y,\Omega _{\gamma }=0)$. Here, we choose $\Omega _{\gamma }$. Note that the critical point *A*(1, 0) and $B(-\,1,0)$ are unstable node i.e. source for $(n=2,\alpha =0.001,\lambda =0.5)$ and the critical point *C*, *D* are the stable solutions i.e. attractor solutions in the given phase plot.
- When $0< \lambda ^{2}< 3-\frac{3\lambda ^{3}(2n+1)}{(n+2)^{2}},\lambda ^{2} < \frac{(n+2)^{2}}{(2n+1)}$; the scalar field prevalent solutions (Points *C* and *D* in Table 1) are the late-time attractor (see Figs. 3, 4 and 7), although when $\frac{3(n+2)^{2}}{2n+1}< \lambda ^{2} < \frac{6(n+2)^{2}}{(2n+1)}$, the solutions are saddle node (see Figs. 5 and 6). There exists an accelerating expansion of the Universe near the critical points *C* and *D* when the value of $\lambda ^{2}$ is less than 2.
- Critical points *E* and *F* are non-hyperbolic for all values of $\lambda$ and $\alpha > 0$ and they are the union of DE and DM. They are the solutions whose acceleration or deceleration is solely dependent on the coupling parameter $\alpha$. It is difficult to examine the stability criteria for these critical points using the linear stability theory.
- The critical points *G* and *H* are non-hyperbolic for $\alpha \in (-\,3,0)$ and they both are the union of DE and DM. They are uniformly decelerating (see Table 2). We’ll now look into the model’s classical stability. Speed of sound $(C_{s})$ plays a significant impact in defining classical stability in cosmo perturbation. When $C^{2}_{s}$ is positive, classical fluctuations may be regarded as stable. Furthermore, $C^{2}_{s}$ must be less than unity, otherwise, signals may be sent along lines like those of the Universe in space. The current stable state of the Universe is as follows
  29 $$\begin{aligned} C^{2}_{s} = 1+\frac{\sqrt{6(2n+1)}xy^{2}\lambda }{(n+2) \left[ 3x^{2}+\frac{\alpha }{2}\Omega _{\gamma }\right] } \end{aligned}$$

Hence, for classical stability (consider $\alpha > 0$)

30 $$\begin{aligned} (n+2)\left[ 6x^{2}+\alpha \Omega _{\gamma }\right] + 2\sqrt{6(2n+1)}xy^{2}\lambda \ge 0 \end{aligned}$$

**Table 5:** Criteria for stability at critical points.

Point	*x*	*y*	$\Omega _{\gamma }$	Local stability	Classical stability
*A*	1	0	0	Unstable	Stable
*B*	1	0	0	Unstable	Stable
*C*	$\frac{\lambda \sqrt{2n+1}}{\sqrt{2}(n+2)}$	$\sqrt{1-\frac{\lambda ^{2}(2n+1)}{2(n+2)^{2}}}$	0	Stable node if $\lambda < \frac{\sqrt{2}(n+2)}{\sqrt{2n+1}}$ Stable point if $\lambda < \frac{\sqrt{\gamma }(n+2)}{\sqrt{2n+1}}$	Stable if $\lambda < \frac{\sqrt{2}(n+2)}{\sqrt{2n+1}}$
*D*	$\frac{\lambda \sqrt{2n+1}}{\sqrt{2}(n+2)}$	$-\sqrt{1-\frac{\lambda ^{2}(2n+1)}{2(n+2)^{2}}}$	0	Stable sink if $0< \alpha < 3-\frac{3\lambda ^{2}(2n+1)}{(n+2)^{2}}$	stable if $\lambda ^{2} \ge 3$
*E*	$-\frac{(\alpha -3)(n+2)}{3\sqrt{2}\lambda \sqrt{2n+1}}$	$\sqrt{1-\frac{(n+2)^{2}(\alpha -3)^{2}}{18\lambda ^{2}(2n+1)}}$	$\frac{(3-\alpha )}{\gamma }\bigg [\frac{1}{3}-\frac{(3-\alpha )(n+2)^{2}}{9\lambda ^{2}(2n+1)}\bigg ]$	Linear stability fails	Stable if $\alpha \ge 3$
*F*	$-\frac{(\alpha -3)(n+2)}{3\sqrt{2}\lambda \sqrt{2n+1}}$	$-\sqrt{1-\frac{(n+2)^{2}(\alpha -3)^{2}}{18\lambda ^{2}(2n+1)}}$	$\frac{(3-\alpha )}{\gamma }\bigg [\frac{1}{3}-\frac{(3-\alpha )(n+2)^{2}}{9\lambda ^{2}(2n+1)}\bigg ]$	Linear stability fails	Stable if $\alpha \ge 3$
*G*	$\sqrt{\frac{-\alpha }{3}}$	0	$1+\frac{\alpha }{3}$	Unstable(saddle)	Unstable
*H*	$-\sqrt{\frac{-\alpha }{3}}$	0	$1+\frac{\alpha }{3}$	Unstable(saddle)	Unstable

The conditions for model reliability at the critical points (shown in Tables 1 and 2) when *x*, *y*, and $\Omega _{\gamma }$ take the corresponding values at the equilibrium points will be discussed next. Tables 1, 3 and 5, we can conclude that *A* and *B* are not locally stable (unstable node or saddle node), and they act like source for $\lambda \in \bigg (-\frac{\sqrt{2}(n+2)}{\sqrt{2n+1}},\frac{\sqrt{2}(n+2)}{\sqrt{2n+1}}\bigg )$ and they correspond to classical stability only. The critical points *C* and *D* correspond to the classical stability of the model if $\lambda ^{2} \ge 3$ and they are identical in all respects. From the perspective of local stability analysis, we observe that the critical points *C* and *D* are stable when $\lambda ^{2} < \frac{(n+2)^{2}}{(2n+1)}$. Critical points *E* and *F* are also classical stable when $\alpha \ge 3$(from Table 5). At the end, from Tables 2, 4 and 5, we conclude that models at the critical points *G* and *H* are not locally stable(saddle) and they are not even classical stable.

## Critical points and cosmological significance

We will now analyze the behavior of the model at the critical points from the phase space analysis of our cosmological model. From Table 1, the values of *A* and *B* show that the scalar field’s kinetic energy entirely dominates the Universe and that acceleration is not feasible. They are not particularly interesting in the current scenario since they relate to a flat, a non-accelerating and unstable Universe without of matter while critical points *C* and *D* are more engaging from the perspective of cosmology. The DE model from the quintessence epoch predicts an accelerated expansion of the cosmos close to *C* and *D*, and both the equilibrium points and the model are locally stable. It should be noted that these critical points line up with cosmological explanations for the recently discovered Universe-wide acceleration in late time. Thus, in the context of this quintessence scalar field, our model predicts the proper evolution scheme for DE density. Critical points *E* and *F* are the combination of both DE and DM. And the critical points *G* and *H* are identical in all aspects and their solutions are ascendant by the kinetic energy of the scalar field, while for *G* and *H*, there exist decelerating Universe.

## Conclusion

With the applications in a wide range of disciplines, including biology, epidemiology, climate and assertions, and of course physics, dynamical systems techniques offer a very potent mathematical technique. It enables us to construct the same system as a collection of non-linear first order ordinary differential equations from a complex set of higher order non-linear differential equations by selecting appropriate new variables. Once the new variables have been analyzed and fixed points for this new system has been found, it will be simple to establish whether or not the fixed point is linearly stable. It provides a geometrical method for understanding a complicated system of equations and for analyzing the fundamental characteristics of the model. Here, we accomplish a dynamical system scrutiny of the Bianchi type I model in the account of barotropic fluid with scalar field in the form of exponential potential. We also discuss the stability and phase space analysis of interacting DE by introducing an interaction between DE and DM of the Universe. In Table 1, we show the relevant critical points of the transformation equation and the values of the physical parameters when the coupling parameter is positive. And when the coupling parameter is negative, we also calculate the critical points which is shown in Table 2. We find out the stable and unstable solutions based on phase space analysis and stability analysis of the equilibrium points. In section “Stability analysis of our cosmological model”, we discuss the linear and classical stability of our cosmological model. We also plotted the phase-space diagrams for different values of $\alpha , \lambda$ while taking the value of $\gamma =4/3$ which are shown in Figs. 1, 2, 3, 4, 5, 6 and 7. And in Fig. 8, we plotted the phase-space diagram for the negative decoupling parameter. From Table 1, the solution at *C* and *D* are governed by scalar field and explains the late-time acceleration. We therefore conclude that, despite the fact that the equilibrium points *C* and *D* correspond to stable model configurations, they are unstable critical points locally. The dimensionless variables included in Eqs. (18)–(20) should be emphasized as not being appropriate for a thorough investigation. The Poincare projection technique can be used to evaluate static solutions that map to infinity-bound critical points, but in bouncing scenarios the expansion rate may cross zero and the state space become non-compact. Therefore, it is preferable to select suitable compact variables for future work to take care of expansions, collapses, static solutions and bounces as well.

## Data Availability

The data-sets generated or analyzed during this study are included in this article.
